# A Method for Assessing the Potential of Multifunctional Retrofitting of Rural Roofs Based on GF-2 Remote Sensing Imagery

**DOI:** 10.3390/s25030770

**Published:** 2025-01-27

**Authors:** Junqi Wang, Linlin Cheng, Yang Zheng, Huizhen Cui, Mengyao Zhu

**Affiliations:** School of Geoscience and Surveying Engineering, China University of Mining and Technology (Beijing), Beijing 100083, China; bqt2100204046@student.cumtb.edu.cn (J.W.); bqt2100204056@student.cumtb.edu.cn (Y.Z.); bqt2200204049@student.cumtb.edu.cn (H.C.); bqt2300204051@student.cumtb.edu.cn (M.Z.)

**Keywords:** rural roofs, roof retrofit, electricity generation, carbon benefits

## Abstract

Green roofs and photovoltaic (PV) roofs are important forms of roof retrofitting, and unused rural roofs provide favorable conditions for the development of green roofs and PV roofs. Here, this study proposes a new method for assessing the potential of multifunctional retrofitting of rural roofs. Firstly, rural roof types were classified into three categories based on GF-2 imagery: flat roofs, east–west pitched roofs, and north–south pitched roofs. The roof types were extracted based on the revised U-Net model, which aims to enhance the extracted features of the buildings and improve the perception of the buildings. Secondly, three types of retrofits—PV roofs, green roofs, and PV-green roofs—were designed taking into account the type, orientation, and area of the roofs. Finally, the potential electricity and carbon benefits of the different retrofit types of roofs were calculated separately, with the aim of realizing an assessment of the potential for roof retrofitting in the rural areas of Mentougou, Beijing. The results of the study showed that 35,407 (281.97 ha) roofs could be used for multifunctional retrofitting. If rural roofs are retrofitted with multifunctionality according to the methodology of this paper, they can absorb an additional 4.66 × 10^4^ kg/yr of CO_2_ and increase biomass production by 0.99 × 10^4^ kg/yr compared to retrofitting only PV roofs, and they can generate an additional 34.1 GWh/yr of electricity and reduce CO_2_ emissions by an additional 3.3 × 10^7^ kg/yr compared to retrofitting to both PV roofs and green roofs. The assessment methodology of this study provides decision makers with data references on the multifunctional potential of rural rooftops for retrofitting, which can optimize the use of rural rooftops, and at the same time is important for promoting the energy transition in rural areas.

## 1. Introduction

The vigorous development of new energy is an important support for promoting green low-carbon development and accelerating the construction of ecological civilization, as well as an important initiative for addressing climate change and fulfilling international commitments [1,2]. Strictly controlling CO_2_ emissions to minimize greenhouse gas input has become a consensus choice for countries around the world [3]. With the development of China’s rural economy, the problems of clean energy use, carbon emission, and environmental protection have become more and more prominent, and the implementation of China’s rural revitalization strategy and the ‘Carbon Peak, Carbon Neutral’ strategy provides an opportunity to solve these problems [4,5]. Rural areas are dispersed and small in scale, with sufficient rooftop resources, which are particularly suitable for energy transition and green energy development [6]. Rooftop photovoltaic (PV) [7,8] and green roofs [9,10], as important forms of green and low-carbon energy development, have been widely used in roof retrofits. Rural areas are weak in fossil energy but have high energy consumption. PV roofs make full use of solar energy resources and enhance energy security while reducing energy consumption and carbon dioxide emissions [11]; green roofs reduce building temperatures and absorb carbon dioxide, and both types of retrofitting are important measures to combat climate change [12,13]. However, there are differences in the retrofitting methods of different roof types, and in order to rationally utilize the roofs, different types of roofs need to be identified and extracted, and then the potential of roof retrofitting needs to be assessed.

The emergence of high-resolution remote sensing images and the development of deep learning technology makes it possible to work on large-scale and fast recognition of building roofs, which reduces the cost of building extraction to a certain extent and can meet the timeliness of building extraction. In existing research, deep learning methods are mostly used for rural roof extraction, and the extraction accuracy of roofs is improved by model improvement. For example, Wang et al. proposed an improved DeeplabV3+ segmentation algorithm for rural roof extraction and applied it to rural rooftop solar photovoltaic [14]. Shi et al. improved it based on the U-Net model and applied it to the publicly available rural blue roof dataset [15]. Sun et al. used a two-phase CNN model to reduce the complexity of the background and improve the detection of rural building accuracy [16]. Zhou et al. used the Efficient Deep Spatial Attention Network (EDSANet) in conjunction with UAV images for extraction [17]. However, none of the above studies focused on the extraction of roof types, and the existing extractions of rural roof types either use empirical values to statistically classify the number of roof types or there are problems in the construction of the dataset. Yu et al. calculated the area of different types of roofs on the basis of the extraction results of the rural buildings based on the frequency of different types of roofs in the study area [18]. Yang et al. constructed a dataset of rural roof types based on UAV images and used the improved Mask R-CNN to extract them [19], and Sun et al. used the improved U-Net model based on Google images to extract roof types [20]. In recent years, SAR imagery has attracted more and more scholars’ attention with its advantages of all-day operation and not being affected by weather factors [21]. Although some scholars have proposed improved methods to increase the resolution of SAR images [22,23], they have not been effectively applied in rural building extraction due to their strong interference from surrounding features and large amounts of data. Although the above scholars have conducted research on rural roof types, it is difficult and time-consuming to acquire UAV data on a large scale, and Google imagery suffers from inconsistent acquisition time and uneven distribution of image patches [24], making it difficult to generalize the data.

The development of high-resolution remote sensing imagery and deep learning technology provides preliminary data support for roof retrofitting, and the research for roof retrofitting methods is also gaining more and more attention from scholars. In existing studies, roof retrofitting is mostly in a single form such as PV roofs or green roofs. Yuliani et al. conducted a study on the development of rural green roofs based on a questionnaire, and the results indicate the need to optimize the role of the community as a means of increasing public awareness of green roofs and thus promoting their development [25]. Liu et al. developed a prioritization index that considered the green space needs of urban functional areas and the suitability of green roofs as a means of quantifying the urban functional areas that are most in need of and suitable for green roof implementation [26]. Hamid et al. used a literature review and questionnaire surveys to find that the main barriers to green roof implementation in Malaysian cities are construction costs, maintenance costs, etc., and based on the reasons for these priorities to effectively develop green roof implementation strategies [27]. Baek et al. determined the appropriate green roof area based on building type and classified green roof types into low, medium, and high management based on green roofs [28]. Hong et al. assessed the potential of green roofs based on the existing and planned buildings, established the indicators of green roof implementability based on the rooftop attributes, and established a strategy for the implementation of the prioritization strategy for green roofs [29]. Aleksejeva et al. classified the available space for green roofs through a GIS approach and assessed the potential of green roofs for subjective well-being, air pollution mitigation, and biodiversity [30]. For the study of PV roofs, a number of scholars have focused on the assessment of roof PV potential around building density, land use data, roof type, etc. Izquierdo et al. estimated the usable area of roofs and PV potential in Spain based on representative cities and a number of readily available data such as land use, population, and building density [31]. Ghaleb et al. calculated PV potentials for utilization efficiency and PV power generation potential for different commercial building types [32]. Tian et al. calculated the total roof area of cities based on open-source satellite data and evaluated their PV power generation potential [33]. Liu et al. evaluated the PV potential of rural roofs using a method that utilizes three-dimensional building models, and their results are important for the development of rural renewable energy, providing a database for solar PV policy development and an important initiative for decision makers to combat climate change [7]. Sun et al. proposed an improved method of installing rooftop photovoltaic panels based on idle rural roofs, obtaining the distribution of PV potential at the village and town scales in rural northern China, and their approach to rural rooftop PV potential can promote the rural energy transition and accelerate the development of clean energy, and their results provided information on the distribution of PV potential for policy makers [20].

Although many scholars have conducted research on roof retrofitting, most of them only focus on PV or green roofs, and there are few studies on multifunctional roof retrofitting. The combination of green roofs and photovoltaic roofs can achieve the dual purpose of roof transformation, whereby on the one hand, solar energy can be used to replace fossil energy to promote energy transition and thus reduce carbon emissions, and on the other hand, the area of green vegetation can be increased to increase the absorption of carbon. Arenandan et al. evaluated the performance of green roofs and PV systems in conjunction with each other and found that green roofs can promote the power generation efficiency of rooftop PV under certain PV installation conditions [34]. Therefore, assessing the potential of green roofs and PV roofs is important for multifunctional roof retrofits. Pan et al. combined PV systems with urban green roofs and proposed a method for assessing the multifunctional retrofit potential of urban rooftops by integrating roof types, roof attributes, and solar attributes in a way that can improve urban sustainability [35]. However, research on roof-based multifunctional retrofitting is limited and focused on urban roofs.

Considering the above research shortcomings, this study proposes a method for assessing the multifunctional potential of rural roofs by using GF-2 imagery, which has a high resolution and maintains spectral information, as a data source, and the U-Net model, which is scalable and adaptable in small sample data, as a base model. The assessment method designs PV roofs, green roofs, and PV-green roofs according to the roof types and calculates the potential power generation and carbon benefits of the multifunctional roof retrofitting, respectively. The main contributions of this study are as follows: (1) a multifunctional retrofit potential assessment method is proposed for rural roofs, which is rare in previous studies of rural roof retrofits; (2) based on GF-2 remote sensing imagery, a revised U-Net model is used to identify rural roof types and count the number and the available area of roofs that can be used for PV roofs, green roofs, and PV-green roofs, respectively; (3) for PV roofs, green roofs, and PV-green roofs, different PV panel installation methods and vegetation planting methods were designed with the aim of obtaining potential power generation and carbon benefits.

## 2. Study Area and Data

**Figure 1:**
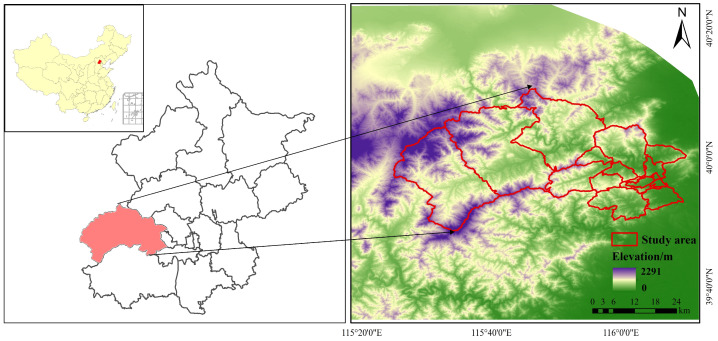
Schematic diagram of the study area.

The Mentougou area is located in the western part of Beijing, China, as shown in Figure 1, at longitude 115°25′–116°10′ E and latitude 39°48′–40°10′ N. The total area of the area is 1448.82 km^2^, and Mentougou is rich in solar energy resources, with an average of about 2470 sunshine hours per annum and an average of 4600–5700 MJ/m^2^. Mentougou belongs to the ecological conservation and development area, and the development of green energy and renewable energy is recommended according to the functional position of the Mentougou district. Therefore, the Mentougou District of Beijing was selected as the study area for this study to assess the potential for multifunctional retrofitting of green roofs and PV roofs on rural roofs.

The experimental data source is the GF-2 image of Mentougou District in Beijing, which contains a 1 m resolution panchromatic image and a 4 m resolution multispectral image. As professional remote sensing image processing software, ENVI has powerful functions and advantages in data preprocessing; therefore, this paper adopts ENVI 5.3 to preprocess the images with radiometric calibration, atmospheric correction, ortho-correction, and image fusion, so as to make full use of the high resolution of the panchromatic images and the spectral information of the multispectral images. Considering the limited computer processing capacity, the image is cropped to 1024 pixels × 1024 pixels in this paper. Before the training of the deep learning model, different roof types need to be labeled. At the same time, rotation and flipping, as data augmentation methods, satisfy the amount of data while only changing the appearance form of the image and do not change the main content and semantic information of the image. Therefore, in this paper, 90-degree rotation, 180-degree rotation, 270-degree rotation, horizontal flipping, and vertical flipping are used for data augmentation as a way to improve the generalization ability of the model, reduce model overfitting, and enhance the robustness of the model.

The annual radiation data were obtained from the PVsyst software, which simulated the annual radiation of the PV panels in the study area with different installation methods. The meteorological data included the daily precipitation, daily maximum temperature, and daily minimum temperature in 2021–2023, which were obtained from the National Center for Meteorological Information (https://data.cma.cn/, accessed on 10 November 2024), and the average of three years was taken as input data.

## 3. Methods

**Figure 2:**
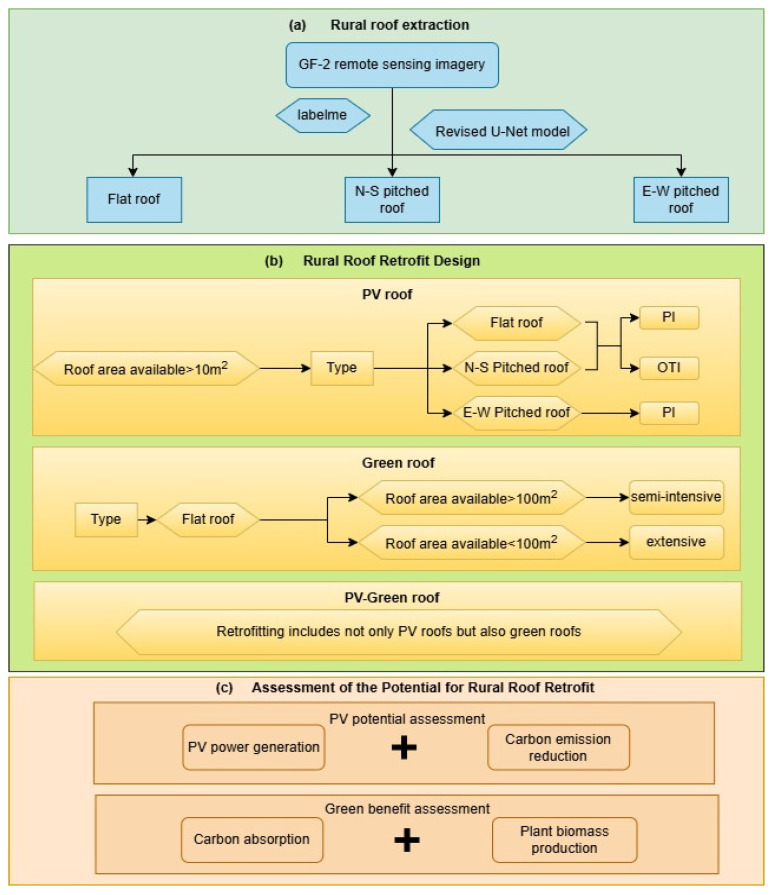
Multifunctional retrofit potential assessment flowchart.

The assessment method proposed in this paper consists of three main steps: (1) Firstly, a dataset of different types of roofs is produced based on GF-2 remote sensing imagery and extracted based on the revised U-Net model for the building roofs in the study area. (2) Three types of roof retrofitting were designed by considering the orientation, slope, and area of the roof: PV roofs, green roofs, and PV-green roofs. The PV roofs are designed for both parallel installation (PI) and optimal tilt installation (OTI), and green roofs are dominated by extensive and semi-intensive vegetation. Lastly, (3) the retrofit potentials of different roof retrofit methods were evaluated, including power generation and carbon emission reductions for PV roofs, carbon dioxide absorption, and plant biomass production for green roofs. The specific process is shown in Figure 2.

### 3.1. Extraction of Different Types of Rural Roofs

#### 3.1.1. Classification and Extraction of Roof Types

Rural roof types are mainly flat and sloped roofs, although there are other types of roofs, such as arched roofs, conical roofs, semi-circular roofs, etc., but none of these types of roofs are suitable for PV retrofit and green roof retrofit, so in this paper, we mainly extract flat and pitched roofs. Flat roofs are roofs with a slope less than 10°, while sloped roofs are roofs with a slope greater than 10° [20,36]. Considering the difference in the absorption of solar radiation between different orientations of pitched roofs, pitched roofs are categorized into north–south (N-S) pitched roofs and east–west (E-W) pitched roofs according to their orientation based on the research study of rural roofs.

**Figure 3:**
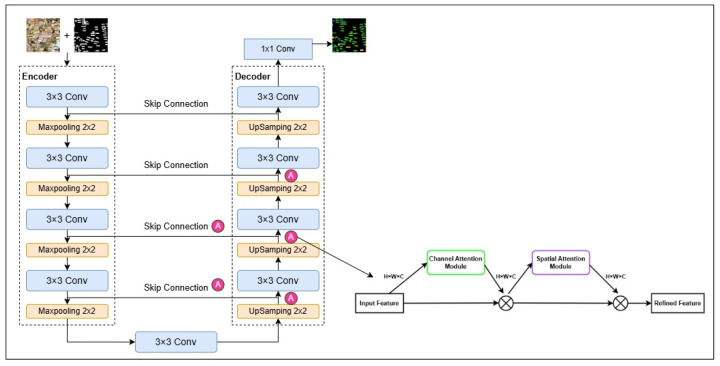
Diagram of revised U-Net model framework.

In this study, different types of rural roofs are extracted based on the U-Net model, which was proposed by Ronneberger in 2015 [37]. The traditional U-Net model adopts an encoder–decoder structure, which performs better and faster segmentation with a small number of samples compared to the traditional network and is widely used in many fields. As shown in Figure 3, it consists of Encoding on the left, Skip Connection in the middle, and Decoding on the right. The encoder extracts four different scales of feature maps through four sub-modules with the same structure, each of which contains a convolutional layer, a ReLU activation function, and a maximum pooling layer. The Skip Connection is responsible for passing these four feature maps to the decoder. The decoder is symmetric to the encoder and integrates the shallow feature maps output from the corresponding encoding layer to supplement the detailed features of the image and restores the resolution of the feature maps layer-by-layer by upsampling to generate the segmentation results. The resolution of the feature maps is recovered layer by layer through up-sampling to generate the segmentation results. U-Net connects different scale feature maps in the channel dimension with a better feature fusion effect, and U-Net is chosen as the base structure for rural roof extraction in this study.

In order to improve the extraction accuracy of the U-Net model, the model was improved in this study. Firstly, the model introduces the CBAM attention mechanism [38]. The main goal of CBAM is to improve the model’s perceptual capabilities by introducing channel attention and spatial attention, thus without increasing the complexity of the network improving performance. The CBAM attention mechanism first performs the average pooling and maximum pooling operations on the input feature maps in the channel dimensions to obtain two different spatial feature maps, and the spatial feature maps are spliced and then passed through a convolutional layer for feature fusion and dimensionality reduction to obtain the spatial attention weight map. In order to prevent excessive learning caused by too many attentional mechanisms, this model only adds attentional mechanisms at the first two jump connections and the first three upsampling processes. Meanwhile, in order to enhance the building features and building edge extraction accuracy, the extraction results of the U-Net model based on the attention mechanism in this study are combined with the original image to form a four channel, which is again extracted by the model with the addition of the attention mechanism. The original feature extraction network can only accept RGB three-channel images, so this study extends the first layer structure to accept the multi-band structure and uses GDAL 3.2.3. to read the images.

#### 3.1.2. Evaluation Metrics

In order to evaluate the extraction accuracy of the improved model for different types of roofs in rural areas, this study adopts three metrics, Recall, *mF*1, and *mIoU*. Recall refers to the proportion of data whose prediction is positive samples and are indeed positive samples to all the indeed positive samples proportion; *F*1 is a comprehensive metric, and where both precision and recall are high, the *F*1 is correspondingly high; *IoU* is the ratio of the intersection of detection results and true values to their concatenation, a metric that is usually lower and more sensitive to misclassification than directly calculating the probability of correct classification for each pixel. Its specific expression is as follows.

(1) $$Recall=\frac{TP}{TP+FN}$$

(2) $$m-F1=\frac{\sum_{i}^{n}\frac{2TP}{FP+2TP+FN}}{n}$$

(3) $$m-IoU=\frac{\sum_{i}^{n}\frac{TP}{TP+TN+FP}}{n}$$

where *TP* denotes true positive, i.e., predicted as a positive sample and predicted correctly; *TN* denotes true negative, i.e., predicted as a negative sample and predicted correctly; *FP* denotes false positive, i.e., predicted as a negative sample but predicted incorrectly; and *FN* denotes false negative, i.e., predicted as a negative sample but predicted incorrectly.

### 3.2. Roof Retrofit Methods and Calculation of Available Area

#### 3.2.1. Definition of Roof Retrofit Methods

**Figure 4:**
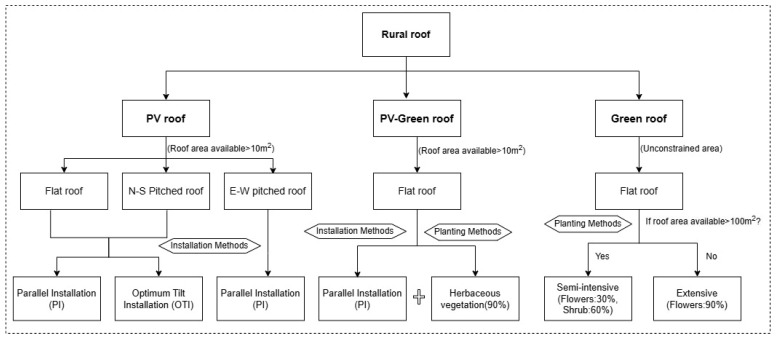
Schematic of the three types of roof retrofits.

In this study, three types of retrofitting methods, namely PV roofs, green roofs, and PV-green roofs, were designed based on Pan et al. [35] taking into account the type, orientation, and area of rural roofs. Figure 4 illustrates the three types of retrofits.

PV roofs refer to the installation of PV panels on rural roofs to directly convert solar radiant energy into electrical energy by utilizing the photovoltaic cell’s photovoltaic effect. The installation of PV on rural roofs requires consideration of maintenance distance, PV conversion efficiency, and operational efficiency, which are set to 0.5 m, 16%, and 85%, respectively, according to monocrystalline silicon panels and related studies [20,39,40,41]. In this paper, two types of PV panel installation are designed: parallel installation (PI) and optimal tilt installation (OTI). The PI method means that the PV panels are installed at an angle parallel to the roof, and the OTI method means that the panels are installed by calculating the tilt angle at the time of maximum annual radiation combined with the slope of the roof.

Green roofs refer to the planting of vegetation on rural roofs to absorb carbon dioxide and produce biomass. Common types of vegetation planting include intensive, semi-intensive, and extensive. The intensive type mainly refers to the planting of some tall shrubs, trees, crops, etc.; the semi-intensive type mainly involves the planting of flowers, shrubs, etc., and is usually designed to have 30% flowers and 60% shrubs; and the extensive type mainly involves the planting of flowers, mosses, and some herbaceous plants. Considering the structure of rural roofs, this study mainly focuses on planting rough and semi-intensive vegetation.

A PV-green roof refers to considering both the PV system and planting vegetation on rural roofs, and in order to minimize the impact between the PV panels and the vegetation, the PV panels are usually installed using the PI method and supported on vegetation by brackets [42], and the vegetation will be chosen to be some shorter and simpler types, such as mosses or herbaceous plants [43,44]. This study was designed to cover 90% of the planted area.

#### 3.2.2. Calculation of Available Area for Different Roof Retrofit Methods

The area available for different roof retrofit methods is influenced by factors such as roof type and solar exposure. PV roof retrofits generally apply to both flat and pitched roofs, and according to previous studies, PV installation not only needs to consider the area of PV panels but also reserve the distance for maintenance in the later stage, so this paper sets the threshold of the roof area for installing PV to be greater than 10 m² [35,45,46]. In addition, the installation area of PV panels is affected by the type of roof. Flat roofs are negligibly affected by roof slope, so the installation area is the extracted projected area of the building minus the maintenance area. Pitched roofs need to consider the influence of roof slope, so the effective area of mountable PV panels needs to be calculated from the projected area and roof slope. Due to the factor of solar irradiation, the installation of PV panels is generally not considered for north-side roofs in northern regions. Considering the safety factor of PV installation, generally E-W pitched roofs are only considered for PI methods.

**Figure 5:**
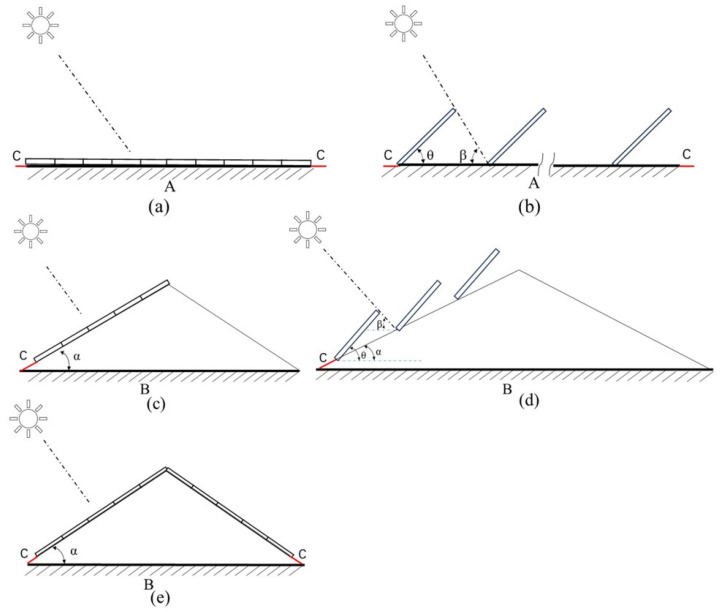
Installation of PV panels on different roof types. (**a**) PI method on flat roofs; (**b**) OTI method on flat roofs; (**c**) PI method on N-S pitched roofs; (**d**) OTI method on N-S pitched roofs; (**e**) PI method on E-W pitched roofs.

Figure 5a,b shows the PV installations for PI method and OTI method on flat roofs, respectively. Figure 5c,d shows the PV installation for N-S pitched roofs with PI and OTI methods, respectively. Figure 5e shows the PV installation for E-W pitched roofs with PI methods, and the available area of PV roofs is calculated as shown in the following equation. Green roofs and PV-green roof retrofits are mainly flat roofs because they require vegetation planting. However, since vegetation planting for green roofs includes both extensive and semi-intensive types, the roof area thresholds for the two planting methods are different, generally planting extensive vegetation on flat roofs smaller than 100 m^2^ and semi-intensive vegetation on flat roofs larger than 100 m^2^, because planting semi-intensive vegetation on roofs of greater than 100 m^2^ has a certain degree of flexibility [35,47]. PV-green roofs need to consider both PV installation and planting vegetation, so generally flat roofs with an area of more than 10 m^2^ are chosen. In order to avoid the impact of PV panels on vegetation, this study only selects PI methods for PV installation in this retrofit method. The available area of the green roof is the area of the flat roof. In the PV-green roof, the installed area of PV panels is the area of the flat roof minus the maintenance area, and the area planted with vegetation is 90% of the area of the flat roof.

(4) S1=(A−2c)(B−2c)

(5) $$S2=\frac{1}{\cos\theta +\sin\theta /\tan\beta }(A-2c)(B-2c)$$

(6) $$S3=(A-2c)(\frac{B}{2\cos\alpha }-c)$$

(7) $$S4=\frac{1}{\cos\theta +\sin\theta /\tan\left(\beta +\alpha \right)}(A-2c)(\frac{B}{2\cos\alpha }-c)$$

(8) $$S5=2\times (A-2c)(\frac{B}{2\cos\alpha }-c)$$

where *S*1 is the area of PV panels installed on flat roofs using the PI method, *S*2 is the area of PV panels installed on flat roofs using the OTI method, *S*3 is the area of PV panels installed on N-S pitched roofs using the PI method, *S*4 is the area of PV panels installed using the OTI method, and *S*5 is the area of PV panels installed on E-W pitched roofs using the PI method. c is the maintenance distance, *θ* is the tilt angle of the PV panels, *β* is the solar altitude angle at 12 o’clock in the winter solstice, and *α* is the roof tilt angle.

### 3.3. Assessment of Roof Retrofit Potential

#### 3.3.1. Calculation of Power Generation Potential and Carbon Reduction Based on PV System

The calculation of power generation potential not only needs to consider the area of installable PV panels on different types of roofs but also needs to estimate the annual radiation per unit area, as shown in Table 1 below. This study simulates the calculation of annual radiation for different types of roofs with different installation modes in the study area in the PvSyst software, and the final PV power generation calculation formula is as follows:

(9) $$E_{i}=S_{i}\times G_{i}\times \sigma \times \mu$$

where *E_i_* is the PV power generation, *S_i_* is the PV panel installation area, *G_i_* is the solar radiation on the roof, *σ* is the PV panel conversion efficiency, and *μ* is the PV panel operation efficiency.

The use of rooftop PV power generation systems will reduce carbon dioxide emissions while making full use of solar energy. According to existing studies, it has been found that power generation through the use of fossil fuels consumes an average of 0.4 kg of standard coal and produces 0.997 kg of carbon dioxide for every 1 KWh of electricity generated [46]. The rooftop PV system also produces 21 g of CO_2_ during its operation [35,48], so the PV system generates 1 KWh of electricity while reducing an additional 0.976 kg of CO_2_. Ultimately, the power generation potential and carbon reduction in the roof-based PV system retrofit can be calculated using the above principles.

#### 3.3.2. Calculation of Carbon Dioxide Absorption and Plant Biomass Based on Green Roofs

**Figure 6:**
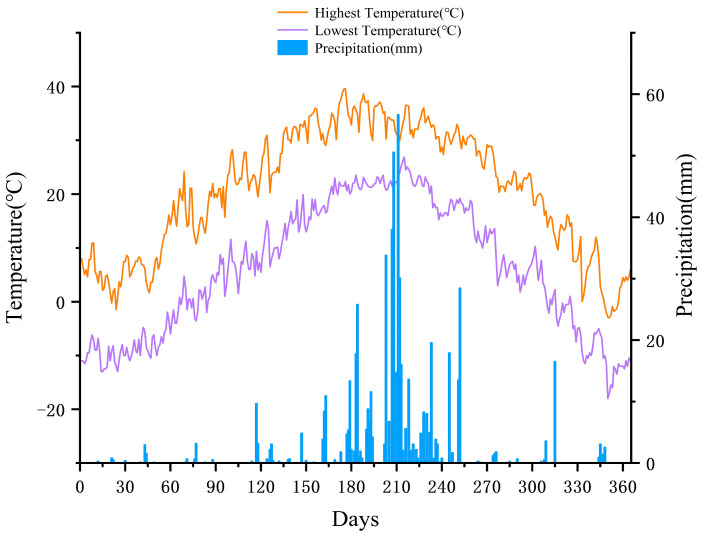
Daily temperature and precipitation.

Green roofs not only absorb carbon dioxide but also produce plant biomass. In this study, the DNDC model was used to simulate the CO_2_ uptake rate and plant biomass production of green roofs in the study area. The simulation of the DNDC model requires the input of three parameters: daily meteorological data (precipitation and maximum and minimum temperatures), soil number parameters, and plant physiological data. The daily meteorological data were averaged from 2021 to 2023, as shown in Figure 6, and the sources of soil parameters and plant physiological data mainly referred to Jing’s setup parameters [49], as shown in Table 2. Its specific soil parameters include the land-use mode, soil texture, irrigation, and so on. The plant’s physiological parameters mainly include the annual N demand, water demand, and optimum temperature. Finally, the CO_2_ uptake rate and plant biomass production were calculated by inputting the above parameters into the DNDC model.

## 4. Results

### 4.1. Extraction Results for Different Types of Roofs

This experiment is based on the Keras deep learning framework using the Windows 11 operating system, with Tensorflow 2.2.0. as the backend and Python 3.7 as the programming language. The graphics card is the NVIDIA GerForce RTX 3060 with 12 GB of video memory. In this paper, the different types of roofs in the study area are extracted based on the revised U-Net model. The extraction results are shown in Table 3 below, which compares the accuracy of the three evaluation indicators of m-IoU, m-F1, and Recall in the revised model and the other extraction models, respectively. As can be seen from Table 3, the revised U-Net model outperforms the other models in all three metrics. Compared with the extraction accuracy of the original model, the revised model improves the m-IoU, m-F1, and Recall by 20.62%, 18.57%, and 12.67%, respectively, and it can be found that the extraction results of the improved U-Net model are better than the original model.

**Figure 7:**
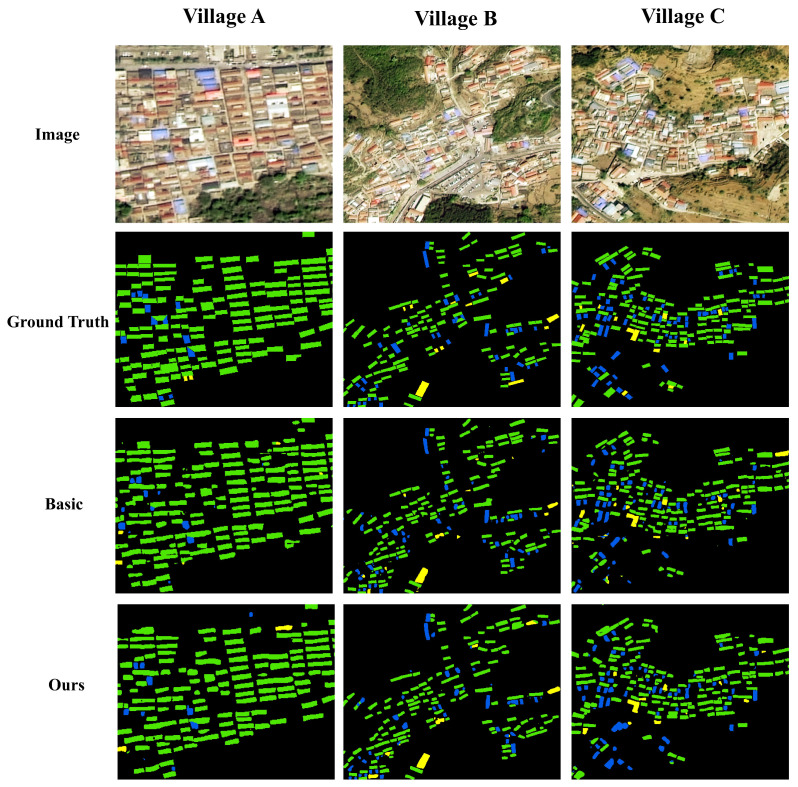
Extraction results using the revised U-Net model.

In this study, three villages, A, B, and C, are taken as examples, and Figure 7 compares the differences between the extraction results using the original model and the improved model with the real labels and the original images, respectively. It can be found that the extracted results have a high degree of similarity with the original images, which can be used in the subsequent analysis of the multifunctional roof retrofitting.

### 4.2. Roof Area Assessment of Different Roof Retrofit Types

The statistics on the extraction results of rural roofs in the Mentougou District, Beijing, showed that there were 281.79 ha (34,554) of roofs, of which 280.5 ha (31,327) could be used for multifunctional retrofitting, which accounted for 99.54% of the entire rural roofs, indicating that there is a large potential for rural roofs to be used for multifunctional retrofitting. Of these, 30,795 roofs can be used for PV roof retrofit, 2202 roofs can be used for green roof retrofit, and 1670 roofs can be used for PV-green roof retrofit. The high number of retrofits to PV roofs and the relatively low number of retrofits to green roofs and PV-green roofs are mainly due to the fact that the majority of roofs in rural areas are pitched roofs, with a low number of flat roofs. As shown in Table 4 below, the available area of the PV panels is smaller than the projected area of the extracted roof, and the available area of PV roofs with the PI method is larger than that with the OTI method. As shown in Table 5 below, 4.92 ha (1529) of the green roofs were used for extensive vegetation and 18 ha (673) were used for semi-intensive vegetation. Moreover, 1670 roofs can be retrofitted with PV-green roofs, of which 18.2 ha of photovoltaic panels can be installed and 20.4 ha can be planted with vegetation.

**Figure 8:**
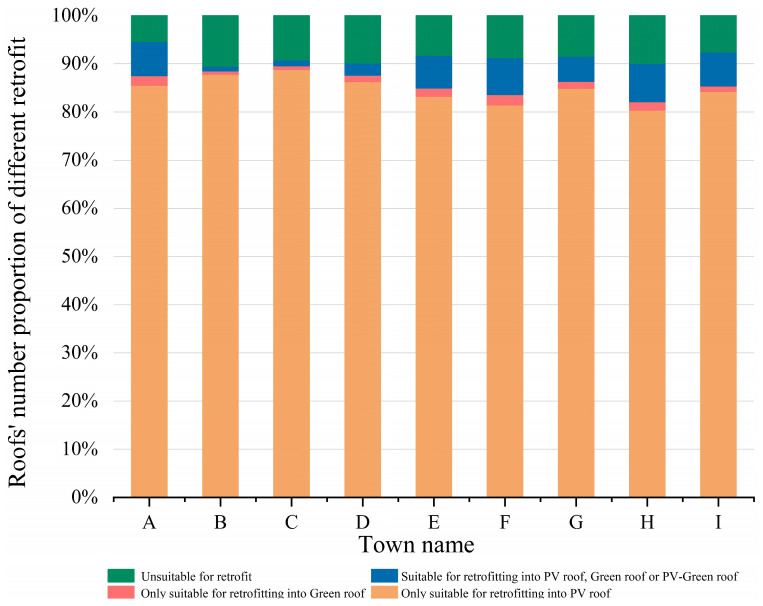
Percentage of the number of roofs with different retrofitting methods.

**Figure 9:**
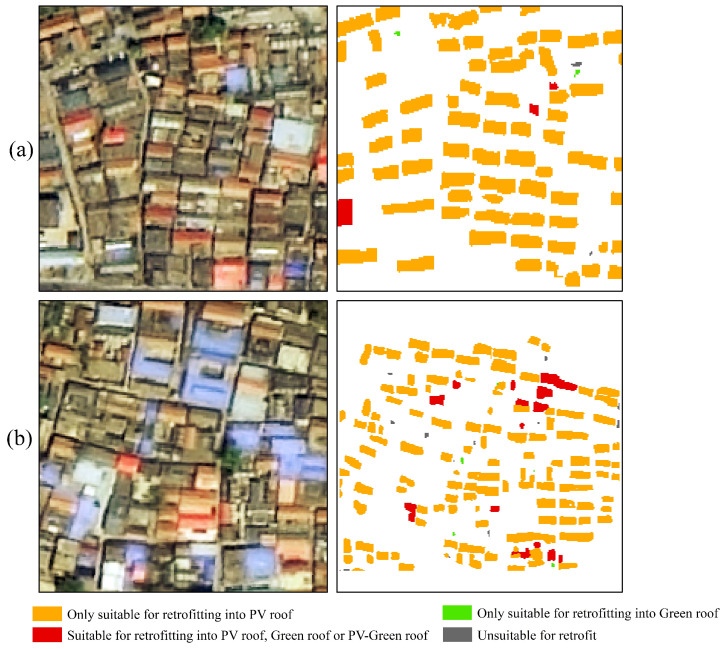
Distribution of different roof retrofitting methods. (**a**,**b**) represent two different retrofitting areas.

Figure 8 shows the proportion of different roof retrofit types in the study area on a town basis, with the largest proportion applicable to PV roof retrofits only, at an average of 83.2%; the average proportions of those unsuitable for retrofitting and those applicable to all three types of roof retrofits were 8.5% and 5.1%, respectively; and the smallest proportion applicable to retrofitting green roofs only was an average of 1.4%. In order to visualize the distribution of the different retrofitting methods more closely, Figure 9 shows the specific details of the potential for roof retrofitting.

### 4.3. Power Generation and Carbon Benefits of Different Roof Retrofit Types

PV roofs are mainly retrofitted from flat roofs, N-S pitched roofs, and E-W pitched roofs; the PV installation methods for flat roofs and N-S pitched roofs include the PI and OTI methods; and the PV installation for E-W pitched roofs is only considered using the PI method. As shown in Table 6, in the PV roof retrofit, when the PV panels are installed in accordance with the PI method for the three roof types, the maximum power that can be generated is 299.695 GWh/year and the maximum reduction in CO_2_ emissions can be 292.502 × 10^6^ kg/year, which indicates that the PV roof retrofit has significant environmental benefits along with a larger power generation capacity. In the PV-green roof retrofit, in order to avoid a large impact on the PV panels and the vegetation, the PV panels are installed using the PI method, and small herbaceous plants are chosen for the vegetation planted, which can generate 34.11 GWh of power generation per year, and CO_2_ emissions are reduced by 33.291 × 10^6^ kg/year. Both the PV-green roof and the PV roof retrofitted from a flat roof were retrofitted from a flat roof under the same conditions (area > 10 m^2^), and the PV installation in the PV-green roof was installed using the PI method, so PV-green roofs produce the same amount of electricity and reduce the same amount of carbon dioxide emissions as the PV roof retrofitted from a flat roof using the PI method.

The annual carbon sequestration and biomass of flowers, shrubs, and herbs were simulated using the DNDC model, and the results, as shown in Table 7, showed that shrubs had the largest CO_2_ sequestration rate (8576.06 kg CO_2_/ha/yr) and biomass production capacity (1785.6 kg CO_2_/ha/yr), followed by flowers and herbs. As shown in Table 8, the CO_2_ uptake of the green roof was 1.641 × 10^5^ kg/yr and the biomass production was 3.37 × 10^4^ kg C/yr, which indicates that the green roof retrofit has significant carbon sequestration and biomass production capacity. The annual CO_2_ sequestration rate of the PV-green roof is 4.53 × 10^4^ kg and biomass production is 9.7 × 10^3^ kg C/year.

## 5. Discussion

The main innovations and contributions of this study are as follows: (1) this study is one of the few assessments of the multifunctional retrofit potential for rural roofs, with most previous studies centering on urban roofs; (2) in response to the lack of reliable and publicly available datasets for rural buildings, this paper constructed a dataset of different types of rural building roofs in the study area based on GF-2 imagery, which was used in subsequent roof retrofits; and (3) combining factors such as roof type, roof orientation, and roof area, this study designed three types of rural roof retrofits—PV roofs, green roofs, and PV-green roofs—and calculated the power generation and carbon benefits of different roof retrofits.

**Figure 10:**
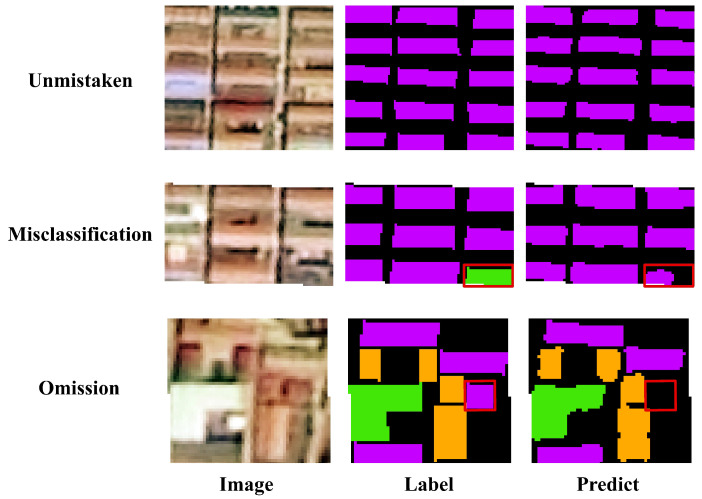
Unmistaken, misclassifications, and omissions in roof extraction.

This study extracts different types of rural building roofs based on a deep learning model, and although the accuracy has been improved to a certain extent, the phenomenon of misclassification and omission still occurs, as shown in Figure 10 below. Although there are misclassification and omission errors in roof extraction, the accuracy of the final extraction result is better compared to the previous extraction accuracy of rural buildings. Corbane et al. used Sentinel-2 satellite images with a resolution of 10 m to predict rural residential areas, and the recall was 80.89% [50]. Liu et al. used Sentinel-2 satellite images with a resolution of 2.5 m to extract rural buildings, and the recall was 78.94% [24]. While the recall of roofs of rural buildings extracted by GF-2 in this paper was 88.53%, which is greater than both of them, the accuracy is within the permissible range, and it can be used to carry out the subsequent analysis of roof retrofit.

In this paper, different roof retrofit methods are designed based on rural roofs to assess the roof retrofit potential. However, only factors such as the roof type, roof area, and solar radiation were considered, and the three types of roofs extracted based on the deep learning model did not take into account the roofs’ building materials, building use, and load-bearing capacity or the occupant’s willingness to do so; thus, there may have been a retrofit assessment of roofs where there was roof damage and the occupant had no desire for a roof retrofit. In addition, due to the slope of pitched roofs, only flat roofs were retrofitted with green roofs and PV-green roofs in this study, while no green roofs or PV-green roofs were retrofitted on pitched roofs, so the suitability of pitched roofs for vegetation planting should be investigated in subsequent studies.

Since the study area has a relatively large proportion of pitched roofs and a relatively small proportion of flat roofs, pitched roofs dominate the retrofit. Although the proportion of flat roofs is relatively small, the proposed multifunctional roof retrofitting method in this paper is still applicable to the study area, and decision makers can flexibly choose the retrofitting method according to the type of roof. PV roofs and PV-green roofs, and PV roofs and green roofs, do not have much influence on each other, so the large proportion of pitched roofs only indicates that the number of PV roofs retrofitted is large but does not affect the number of green roofs and PV-green roof retrofits. At the same time, the retrofitting approach proposed in this paper is also applicable to other study areas and has implications for roof retrofitting in other rural areas.

For the retrofitting of PV roofs, the PI method used in this study produced more power generation and reduced carbon emissions compared to the OTI method, but the area of PV panels used for the PI method was larger than that of the OTI method, and therefore the installation cost of the PV panels was relatively high. However, since a certain angle is required for the installation of a photovoltaic panel using the OTI method, it is important to take into account the safety hazards that exist during installation. In future studies, the reduction in electricity generation and carbon emissions from PV roofs should be considered along with cost and feasibility, balancing electricity generation and carbon benefits with installation cost and feasibility. In addition, this study ignored the impact between PV systems and vegetation planting in PV-green roof retrofits, but in practice, PV systems can have an impact on vegetation growth due to installation factors [51,52], and at the same time, vegetation can also affect the building’s energy consumption [53]. The impacts of PV roofs and green roofs and respective impacts on buildings should also be taken into account in future studies to refine the assessment of the potential for rural roof retrofitting.

## 6. Conclusions

In this study, a multifunctional retrofit potential assessment method for rural roofs was innovatively proposed. Based on the actual situation in rural areas, this paper extracts flat roofs, N-S pitched roofs, and E-W pitched roofs using the improved U-Net model, and then designs three types of retrofit methods for PV roofs, green roofs, and PV-green roofs for the type of roofs, the slope of the roofs, the orientation of the roofs, and their usable areas, respectively. PV roofs took into account the PI and OTI methods, green roofs adopted extensive planting and semi-intensive planting, the PV system in the PV-green roof was installed using the PI method, and the green system adopted extensive planting. Finally, their retrofitting potentials were evaluated.

Applying the potential assessment method in this paper to the study area, the results show that a total of 31,327 (280.5 ha) roofs can be used for multifunctional retrofitting, of which 30,795 roofs can be used for PV roof retrofitting, 2202 roofs can be used for green roof retrofitting, and 1670 roofs can be used for PV-green roof retrofitting. If only retrofitting into PV roofs can generate a maximum of 299.695 GWh/year, CO_2_ emissions can be reduced by a maximum of 2.93 × 10^8^ kg/year. Using retrofitting to PV roofs and green roofs, a maximum of 265.586 GWh/year can be generated, CO_2_ emissions can be reduced by a maximum of 2.59 × 10^8^ kg/year, CO_2_ absorption is 1.64 × 10^5^ kg/year, and biomass production is 3.37 × 10^4^ kg C/year. If the rural roofs are retrofitted with multifunction, a maximum of 299.695 GWh/year can be generated, CO_2_ emissions can be reduced by 2.93 × 10^8^ kg/year, CO_2_ absorption is 4.66 × 10^4^ kg/year, and biomass production is 0.99 × 10^4^ kg C/year. In summary, this paper provides a research method for rural roof space utilization, and the results of the study provide data support and research solutions for the implementation of multifunctional retrofits of rural roofs, which can promote the implementation of multifunctional retrofits of rural roofs and is of great significance for the creation of green villages.

## Figures and Tables

**Table 1:** Annual solar radiation from different installations.

Roof Type	PI (kWh/(m^2^ꞏYear))	OTI (kWh/(m^2^ꞏYear))
Flat roof	1375.3	1597.2
E-W pitched roof	1313.6	1597.2
N-S pitched roof	1576.7	1597.2

**Table 2:** Soil parameters and physiological parameters of different crop types.

Crop Type	Land-Use	Top Soil Properties	Max. Biomass Production (kg C/ha/yr)	Annual N Demand (kg N/ha)	Thermal Degree Days for Maturity	Water Demand (g Water/g DM)	N Fixation Index (Crop N/N from Soil)	Optimum Temperature (°C)
Herbaceous vegetation	Moist grassland	Sandy clay loam	11(Grain)/275 (Leaf)/ 275 (Stem)/539 (Root)	40.7	1200	300	1	15
Flowers	Moist grassland	Loam	913.5 (Grain)/182.7 (Leaf)/182.7 (Stem)/ 548.1 (Root)	71.5575	1400	800	1	25
Shrubs	Moist grassland	Loam	24 (Grain)/600 (Leaf)/ 600 (Stem)/1176 (Root)	16.64	2000	250	1	15

**Table 3:** Building extraction results using different models.

	m-IoU/%	m-F1/%	Recall/%
Deeplab V3	33.86 ± 0.23	40.99 ± 0.34	47.92 ± 0.48
PSPNet	40.22 ± 1.15	51.07 ± 1.54	48.98 ± 1.95
U-Net	52.33 ± 0.38	64.94 ± 0.39	75.86 ± 1.22
U-Net + CA	52.08 ± 1.17	64.62 ± 1.24	72.05 ± 1.88
U-Net + SE	51.75 ± 1.22	64.27 ± 1.33	72.64 ± 0.1
U-Net + CBAM	55.88 ± 0.55	68.56 ± 0.65	77.36 ± 2.26
Ours	72.95 ± 0.45	83.51 ± 0.32	88.53 ± 1.11

**Table 4:** Available area for different installations of photovoltaic system roof retrofits.

Retrofit Type	Rooftop Type	Projection Area (m^2^)	PV Panel Installation Area (m^2^)	
			PI	OTI
PV roofs	Flat roofs	2.27 × 10^5^	1.82 × 10^5^	9.19 × 10^4^
	N-S pitched roofs	1.99 × 10^6^	8.39 × 10^5^	7.40 × 10^5^
	E-W pitched roofs	5.83 × 10^5^	4.79 × 10^5^	-
PV-Green roofs	Flat roofs	2.27 × 10^5^	1.82 × 10^5^	-

**Table 5:** Available area of green roof retrofits.

Retrofit Type	Plant Type	Projection Area (m^2^)	Available Area (m^2^)
Green roofs	extensive	4.92 × 10^4^	4.43 × 10^4^
	semi-intensive	1.80 × 10^5^	1.62 × 10^5^
PV-Green roofs	extensive	2.27 × 10^5^	2.04 × 10^5^

**Table 6:** Power generation and carbon emission reduction in PV system roof retrofit.

Retrofit Type	Rooftop Type	Electricity Output/ (GWh/yr)		Reduction in Carbon Emissions/ (×10^6^ kg/yr)	
		PI	OTI	PI	OTI
PV roofs	Flat roofs	34.11	19.965	33.291	19.486
	N-S pitched roofs	179.903	160.847	175.585	156.987
	E-W pitched roofs	85.683	-	83.626	-
PV-Green roofs	Flat roofs	34.11	-	33.291	-

**Table 7:** Carbon fixation and biomass modeling results.

	Herbaceous Vegetation	Flowers	Shrubs
Plant photosynthesis (kg CO_2_/ha/yr)	2213.744	7245.681	8576.056
Plant biomass production kg C/ha/yr	473.41	1464.8	1785.6

**Table 8:** Carbon absorption and biomass production from green roof retrofits.

Retrofit Type	Plant Type	Absorption of Carbon Emissions/ (×10^4^ kg CO_2_/yr)	Plant Biomass Production/ (×10^4^ kg C/yr)
Green roofs	extensive	3.21	0.65
	semi-intensive	13.2	2.72
PV-Green roofs	extensive	4.53	0.97

## Data Availability

The datasets presented in this article are not readily available.

## References

[1] Zou C., Ma F., Pan S., Zhao Q., Fu G., Zhang G., Yang Y., Yu H., Liang Y., Lin M. (2023). Global energy transition revolution and the connotation and pathway of the green and intelligent energy system. Pet. Explor. Dev..

[2] Liu Y., Dong X., Dong K. (2023). Pathway to prosperity? The impact of low-carbon energy transition on China’s common prosperity. Energy Econ..

[3] Wang S., Shen Y., Song S., Liu L., Gu L., Wei J. (2023). Change of coal energy status and green and low-carbon development undernthe “dual carbon” goal. J. China Coal Soc..

[4] Zhang X., Barrington-Leigh C.P., Robinson B.E. (2024). Rural household energy transition in China: Trends and challenges. J. Clean. Prod..

[5] Shi Q., Li Z., Xu Y., Yan T., Chen M. (2023). Dynamic Scenario Simulations of Sustainable Rural and Towns Development in China: The Case of Wujiang District. Sustainability.

[6] Dou C., Zuo C., Jia Y., Jiang L., Zheng L. (2023). Analysis on Development Potential of Rooftop Distributed PV Power Generation of Rural Residential Dwellings in China. Sol. Energy.

[7] Liu J., Wu Q., Lin Z., Shi H., Wen S., Wu Q., Zhang J., Peng C. (2023). A novel approach for assessing rooftop-and-facade solar photovoltaic potential in rural areas using three-dimensional (3D) building models constructed with GIS. Energy.

[8] Yang Y., Si Z., Jia L., Wang P., Huang L., Zhang Y., Ji C. (2024). Whether rural rooftop photovoltaics can effectively fight the power consumption conflicts at the regional scale—A case study of Jiangsu Province. Energy Build..

[9] Chen N., Deng Q., Chen Q., Wang Z. (2024). Green roof heat transfer coefficient measurement and impact of plant species and moisture. Energy Build..

[10] Huang J., Kong F., Yin H., Middel A., Liu H., Meadows M.E. (2023). Green roof effects on urban building surface processes and energy budgets. Energy Convers. Manag..

[11] Sailor D.J., Anand J., King R.R. (2021). Photovoltaics in the built environment: A critical review. Energy Build..

[12] Ladenburg J., Kim J., Zuch M., Soytas U. (2024). Taking the carbon capture and storage, wind power, PV or other renewable technology path to fight climate change? Exploring the acceptance of climate change mitigation technologies—A Danish national representative study. Renew. Energy.

[13] Scharf B., Kraus F. (2019). Green Roofs and Greenpass. Buildings.

[14] Wang X., Li D. (2022). Solar Rooftop Segmentation in Rual Areas Based on Improved Deeplabv3+. Comput. Appl. Softw..

[15] Shi X., Huang H., Pu C., Yang Y., Xue J. (2022). CSA-UNet: Channel-Spatial Attention-Based Encoder-Decoder Network for Rural Blue-Roofed Building Extraction from UAV Imagery. IEEE Geosci. Remote Sens. Lett..

[16] Sun L., Tang Y., Zhang L. (2017). Rural Building Detection in High-Resolution Imagery Based on a Two-Stage CNN Model. IEEE Geosci. Remote Sens. Lett..

[17] Zhou J., Liu Y., Nie G., Cheng H., Yang X., Chen X., Gross L. (2022). Building Extraction and Floor Area Estimation at the Village Level in Rural China Via a Comprehensive Method Integrating UAV Photogrammetry and the Novel EDSANet. Remote Sens..

[18] Yu H. (2019). Building Roof Extraction and Solar Energy Potential Assessment Based on High Resolution Remote Sensing Image. Master’s Thesis.

[19] Wang Y., Li S., Teng F., Lin Y., Wang M., Cai H. (2022). Improved Mask R-CNN for Rural Building Roof Type Recognition from UAV High-Resolution Images: A Case Study in Hunan Province, China. Remote Sens..

[20] Sun T., Shan M., Rong X., Yang X. (2022). Estimating the spatial distribution of solar photovoltaic power generation potential on different types of rural rooftops using a deep learning network applied to satellite images. Appl. Energy.

[21] Liu D., Chang S., Deng Y., He Z., Wang F., Zhang Z., Han C., Yu C. (2024). A Novel Spaceborne SAR Constellation Scheduling Algorithm for Sea Surface Moving Target Search Tasks. IEEE J. Sel. Top. Appl. Earth Obs. Remote Sens..

[22] Chang S., Deng Y., Zhang Y., Wang R., Qiu J., Wang W., Zhao Q., Liu D. (2022). An Advanced Echo Separation Scheme for Space-Time Waveform-Encoding SAR Based on Digital Beamforming and Blind Source Separation. Remote Sens..

[23] Chang S., Deng Y., Zhang Y., Zhao Q., Wang R., Zhang K. (2022). An Advanced Scheme for Range Ambiguity Suppression of Spaceborne SAR Based on Blind Source Separation. IEEE Trans. Geosci. Remote Sens..

[24] Liu Z., Tang H., Feng L., Lyu S. (2023). China Building Rooftop Area: The first multi-annual (2016–2021) and high-resolution (2.5 m) building rooftop area dataset in China derived with super-resolutionsegmentation from Sentinel-2 imagery. Earth Syst. Sci. Data.

[25] Yuliani S., Hardiman G., Setyowati E. (2020). Green-Roof: The Role of Community in the Substitution of Green-Space toward Sustainable Development. Sustainability.

[26] Liu W., Qian Y., Yao L., Feng Q., Engel B.A., Chen W., Yu T. (2022). Identifying city-scale potential and priority areas for retrofitting green roofs and assessing their runoff reduction effectiveness in urban functional zones. J. Clean. Prod..

[27] Hamid H.N.A., Romali N.S., Rahman R.A. (2023). Key Barriers and Feasibility of Implementing Green Roofs on Buildings in Malaysia. Buildings.

[28] Baek K.Y., Kim H.G. (2022). Analyzing the Efficiency of Increasing Suitable Habitat Area for *Paridae* by Roof Greening Method Based on Building Type: Case Study of Suwon City, Republic of Korea. Sens. Mater..

[29] Hong W., Guo R., Tang H. (2019). Potential assessment and implementation strategy for roof greening in highly urbanized areas: A case study in Shenzhen, China. Cities.

[30] Aleksejeva J., Voulgaris G., Gasparatos A. (2022). Assessing the potential of strategic green roof implementation for green infrastructure: Insights from Sumida ward, Tokyo. Urban For. Urban Green..

[31] Izquierdo S., Rodrigues M., Fueyo N. (2008). A method for estimating the geographical distribution of the available roof surface area for large-scale photovoltaic energy-potential evaluations. Sol. Energy.

[32] Ghaleb B., Asif M. (2022). Assessment of solar PV potential in commercial buildings. Renew. Energy.

[33] Tian R., Meng E., Shu Y., Li J., Zhou B., Zhao H. (2024). Experimental study of the thermal performance of a new type of PV roof. Int. Commun. Heat Mass Transf..

[34] Arenandan V., Wong J.K., Ahmed A.N., Chow M.F. (2022). Efficiency enhancement in energy production of photovoltaic modules through green roof installation under tropical climates. Ain Shams Eng. J..

[35] Pan Z., Wang C., Yu B., Chen Z., Yuan Y., Li G., Zhang J., Xiao T. (2024). Assessing multifunctional retrofit potential of urban roof areas and evaluating the power and carbon benefits under efficient retrofit scenarios. J. Clean. Prod..

[36] de Vries T.N.C., Bronkhorst J., Vermeer M., Donker J.C.B., Briels S.A., Ziar H., Zeman M., Isabella O. (2020). A quick-scan method to assess photovoltaic rooftop potential based on aerial imagery and LiDAR. Sol. Energy.

[37] Ronneberger O., Fischer P., Brox T. U-Net: Convolutional Networks for Biomedical Image Segmentation. Proceedings of the 18th International Conference on Medical Image Computing and Computer-Assisted Intervention (MICCAI).

[38] Woo S., Park J., Lee J.-Y., Kweon I.S. CBAM: Convolutional Block Attention Module. Proceedings of the 15th European Conference on Computer Vision (ECCV).

[39] Huang Z., Mendis T., Xu S. (2019). Urban solar utilization potential mapping via deep learning technology: A case study of Wuhan, China. Appl. Energy.

[40] Aslani M., Seipel S. (2022). Automatic identification of utilizable rooftop areas in digital surface models for photovoltaics potential assessment. Appl. Energy.

[41] Xu S., Li Z., Zhang C., Huang Z., Tian J., Luo Y., Du H. (2021). A method of calculating urban-scale solar potential by evaluating and quantifying the relationship between urban block typology and occlusion coefficient: A case study of Wuhan in Central China. Sustain. Cities Soc..

[42] Osma G., Ordonez G., Hernandez E., Quintero L., Torres M. The impact of height installation on the performance of PV panels integrated into a green roof in tropical conditions. Proceedings of the 2nd International Conference on Energy Production and Management in the 21st Century—The Quest for Sustainable Energy (EQ).

[43] Chemisana D., Lamnatou C. (2014). Photovoltaic-green roofs: An experimental evaluation of system performance. Appl. Energy.

[44] Lamnatou C., Chemisana D. (2015). A critical analysis of factors affecting photovoltaic-green roof performance. Renew. Sustain. Energy Rev..

[45] Huang Y., Chen Z., Wu B., Chen L., Mao W., Zhao F., Wu J., Wu J., Yu B. (2015). Estimating Roof Solar Energy Potential in the Downtown Area Using a GPU-Accelerated Solar Radiation Model and Airborne LiDAR Data. Remote Sens..

[46] Chen Z., Yu B., Li Y., Wu Q., Wu B., Huang Y., Wu S., Yu S., Mao W., Zhao F. (2022). Assessing the potential and utilization of solar energy at the building-scale in Shanghai. Sustain. Cities Soc..

[47] Cascone S., Catania F., Gagliano A., Sciuto G. (2018). A comprehensive study on green roof performance for retrofitting existing buildings. Build. Environ..

[48] Kim H.C., Fthenakis V., Choi J.-K., Turney D.E. (2012). Life Cycle Greenhouse Gas Emissions of Thin-film Photovoltaic Electricity Generation. J. Ind. Ecol..

[49] Jing R., Liu J., Zhang H., Zhong F., Liu Y., Lin J. (2022). Unlock the hidden potential of urban rooftop agrivoltaics energy-food-nexus. Energy.

[50] Corbane C., Syrris V., Sabo F., Politis P., Melchiorri M., Pesaresi M., Soille P., Kemper T. (2021). Convolutional neural networks for global human settlements mapping from Sentinel-2 satellite imagery. Neural Comput. Appl..

[51] Xia Z., Li Y., Zhang W., Guo S., Zheng L., Jia N., Chen R., Guo X., Du P. (2023). Quantitatively distinguishing the impact of solar photovoltaics programs on vegetation in dryland using satellite imagery. Land Degrad. Dev..

[52] Choi C.S., Macknick J., McCall J., Bertel R., Ravi S. (2024). Multi-year analysis of physical interactions between solar PV arrays and underlying soil-plant complex in vegetated utility-scale systems. Appl. Energy.

[53] Dwijendra N.K.A., Muda I., Milanes C.B., Kumar N.B., Abosinnee A.S., Akhmadeev R. (2023). How do green roofs affect per capita energy consumption in residential buildings under various climate conditions?. Sustain. Energy Technol. Assess..

